# SWI3A/B regulates the transition from vegetative to reproductive phase in the liverwort Marchantia polymorpha

**DOI:** 10.1007/s00497-026-00537-5

**Published:** 2026-05-20

**Authors:** Maria Lozano-Quiles, Parth K. Raval, Stefan A Rensing, Sven B. Gould

**Affiliations:** 1 Institute for Molecular Evolution, Heinrich–Heine–Universität Düsseldorf, 40225 Düsseldorf, Germany; 2 Faculty of Chemistry and Pharmacy, Albert-Ludwigs-Universität Freiburg, 79104 Freiburg, Germany

**Keywords:** *Marchantia*, Reproductive development, Vegetative growth, Alternation of generations, Gametangia, Spermatozoid

## Abstract

Land plants alternate between multicellular haploid and diploid phases, requiring a tight coordination between vegetative growth and sexual reproduction. We investigated *SWI3A/B*, an ancient core subunit of the SWI/SNF complex, of the non-vascular liverwort *Marchantia polymorpha*. A mutation in the promoter of Mp*SWI3A/B* affected gametangiophore development and spermiogenesis in males, revealing its male-specific role in reproductive development. The Mp*SWIA/B* mutant line amplifies vegetative propagation in males under conditions that would normally induce reproductive growth. The phenotype was underpinned by transcriptomic changes, showing that MpSWI3A/B modulates key regulators of gametangiophore initiation (e.g. BONOBO, GLID), sperm development and motility (e.g. DUO1, PKAR), and asexual reproduction (e.g. KAI2). We propose that, as a chromatin-level regulator, MpSWI3A/B may contribute to balancing vegetative and reproductive phases and highlight the protein’s potential when exploring ancient epigenetic functions that coordinate developmental phase transitions in land plants.

**Supplementary Information:**

The online version contains supplementary material available at 10.1007/s00497-026-00537-5.

## Key Message

MpSWI3 regulates male reproductive development by balancing vegetative and sexual phases. The protein coordinates gametangiophore formation, spermiogenesis and vegetative propagation through a chromatin-level control of key developmental genes.

**Supplementary Information:**

The online version contains supplementary material available at 10.1007/s00497-026-00537-5.

## Introduction

Land plants (Embryophyta) exhibit a haplodiplontic life cycle, in which both haploid and diploid phases are multicellular and proliferate through mitosis (Niklas and Kutschera 2010; Bowman et al. 2017; de Vries and Archibald 2018). The haploid gametophyte produces gametes mitotically, whereas the diploid sporophyte produces spores meiotically that develop into new gametophytes. This ‘alternation of generations’ contrasts with the diplontic life cycle of animals and the haplontic cycle of many streptophyte algae, where only the diploid and haploid phase, respectively is multicellular (Niklas and Kutschera 2010; Bowman et al. 2017). Following the transition of the streptophyte algal ancestor of land plants onto land, early plants still depended on flagellated spermatozoids for fertilization. Flagellated spermatozoids were lost twice in land plant evolution, within conifers and angiosperms (Meyberg et al. 2020). Extant gymnosperms such as cycads produce both flagellated sperm and pollen, whereas angiosperms (flowering plants) rely entirely on pollen for fertilization (Renzaglia 2001; Niklas and Kutschera 2010). Bryophytes, which diverged from vascular plants soon after plant terrestrialization, retain flagellated spermatozoids and in contrast to spermatophytes are gametophyte (haploid) dominant (Pandey et al. 2022). As such, they provide a valuable system to study the regulation and molecular control of male reproductive development in plants (Renzaglia 2001).

Reproductive development is orchestrated by genetic and epigenetic regulators that likely date back to the origin of eukaryotes (Goodenough and Heitman 2014; She and Baroux 2014; Speijer et al. 2015). In plants, this regulation involves DNA-binding proteins, chromatin modifiers, and transcription factors that together form networks coordinating developmental transitions through epigenetic and protein–protein interactions (She and Baroux 2014; Borg and Berger 2015). Among these, chromatin-remodeling complexes (CRCs) form a nexus between regulatory signaling and chromatin-based transcriptional control (Clapier and Cairns 2009; Han et al. 2013). SWI3, a key subunit of the SWITCH/SUCROSE NONFERMENTING (SWI/SNF) CRC, modulates nucleosome architecture via H3 acetylation at lysine 27 (H3K27), thereby activating reproductive genes and counteracting PRC2-mediated H3K27 methylation (Sarnowski et al. 2005; Zheng and Chen 2011; Wu et al. 2012). In *Arabidopsis*, SWI3-dependent acetylation of floral identity and reproductive genes (e.g., AP3 and SEP) ensures proper flowering timing and viable seed formation (Molitor et al. 2014; Yan et al. 2019). This activity counteracts H3K27me3-mediated repression that silences key reproductive regulators such as FLOWERING LOCUS C (FLC) and maintains AGAMOUS, APETALA3, and SEPALLATA genes inactive until floral induction (Jiang et al. 2013; Zheng et al. 2019).

In flowering plants, the SWI3 family has diversified into four members forming two phylogenetic groups, SWI3A/B and SWI3C/D. Likely due to an ancestral genome reduction event (Zhang et al. 2020; Donoghue et al. 2021a, b; Harris et al. 2022; Linde et al. 2023), however, bryophytes encode only a single SWI3A/B-type gene (Genau et al. 2021). In the moss *Physcomitrium patens*, SWI3A/B mutants fail to degrade cell-wall layers during spermatogenesis, resulting in impaired sperm motility, incomplete cytoplasmic reduction, infertility, and defective male reproduction (Genau et al. 2021). While SWI3A/B function has been investigated in *Arabidopsis* and *Physcomitrium*, both species are monoecious, (Koornneef and Meinke 2010; Rensing et al. 2020) and possible sex-specific roles of SWI3A/B in gametophyte differentiation, transcriptional regulation, and parental contribution remain therefore uncertain. *Marchantia polymorpha*, a dioecious bryophyte with sex chromosomes determining distinct male and female individuals (Bowman 2016) hence provides a system to address this issue. Furthermore, genes controlling gametangial development are only expressed during gametangiogenesis in *Physcomitrium* (Perroud et al. 2018), but they are already differentially expressed during the vegetative stage in *Marchantia*, hinting at their sex-specific regulation in the liverwort already before its reproductive organs form (Hisanaga et al. 2019).

In *M. polymorpha*, the transition from vegetative growth to reproductive development is triggered by an increase in far-red (FR) light, perceived by the phytochrome MpPHY, which regulates MpPIF (PHYTOCHROME-INTERACTING FACTOR) to initiate gametangiophore formation (Inoue et al. 2019). MpBONOBO (MpBNB) forms heterodimers with LOTUS JAPONICUS ROOTHAIRLESS-LIKE/DEFECTIVE REGION OF POLLEN (LRL/DROP) proteins (MpLRL) in gametophytic nuclei, constituting a conserved bHLH module required for germ cell differentiation and gametangiophore development (Yamaoka et al. 2018; Saito et al. 2023). MpBNB also activates MpGLID (GERMLINE IDENTITY DETERMINANT), promoting the formation of male germ cell-like cells during gametangium development (Ren et al. 2024). MpBNB accumulation in female gamete precursor cell requires the histidine kinase MpCKI1 (CYTOKININ-INDEPENDENT 1) mediated asymmetric cell divisions that establish the archegonial germline (Bao et al. 2024). Gibberellin-related diterpenoid pathway involving MpCPS (ent-copalyl diphosphate synthase) and MpKOL (ent-kaurene oxidase-like (KO-like) cytochrome P450s) are necessary for further FR-dependent gametangiophore development (Sun et al. 2023). Additional transcriptional regulators, including members of the KNOX (KNOTTED-like homeobox)/BELL (BELL-like homeobox transcription factor family) family such as MpKNOX1, MpBELL1, MpBELL4, and MpBELL5, contribute to zygotic development and reproductive cells differentiation (Dierschke et al. 2021). R2R3-MYB transcription factor MpDUO1 (DUO POLLEN 1) is essential for sperm differentiation (Higo et al. 2018), while genes such as MpPKAR (Protein Kinase A regulatory subunit) and a radial spoke protein homolog Mp6g07660, contribute to the formation and motility of the sperm flagella (Yamamoto et al. 2024). While these studies define key signalling and transcriptional regulators of reproductive development in *Marchantia*, the role of chromatin remodelling complexes coordinating these gene regulatory programs remain largely unknown.

Here, we provide data on MpSWI3A/B, suggesting the protein to regulate the transition from vegetative to reproductive growth in male plants and to govern key aspects of sexual development. Mp*SWI3A/B* overexpression mutants reinforce vegetative propagation and display delayed development sexual organ, hinting that MpSWI3A/B may act as an epigenetic control point between these distinct strategies of survival and reproduction.

## Results

### SWI3A/B regulates reproductive development and spermiogenesis in *Marchantia*

Across plants, SWI3A/B is a highly diversified protein with two copies encoding it in *Marchantia* that cluster with the SWIA/B and SWIC/D gene families of *Arabidopsis* (Genau et al. 2021). Their domain analysis validates the presence of MYB/SANT domain characteristic for this protein family (Fig. S1). In addition to this domain, SWI3C/D also contain a zinc finger domain separating it from SWI3A/B. This further confirms Mp8g15610 (aka Mapoly0079s0052) to represent the bryophyte’s SWIA/B homologue (referred to as Mp*SWI3A/B*). We focused on this gene, since SWI3A/B was shown to affect gametangiogenesis in *Arabidopsis*(Sarnowski et al. 2005).

**Fig. 1:**
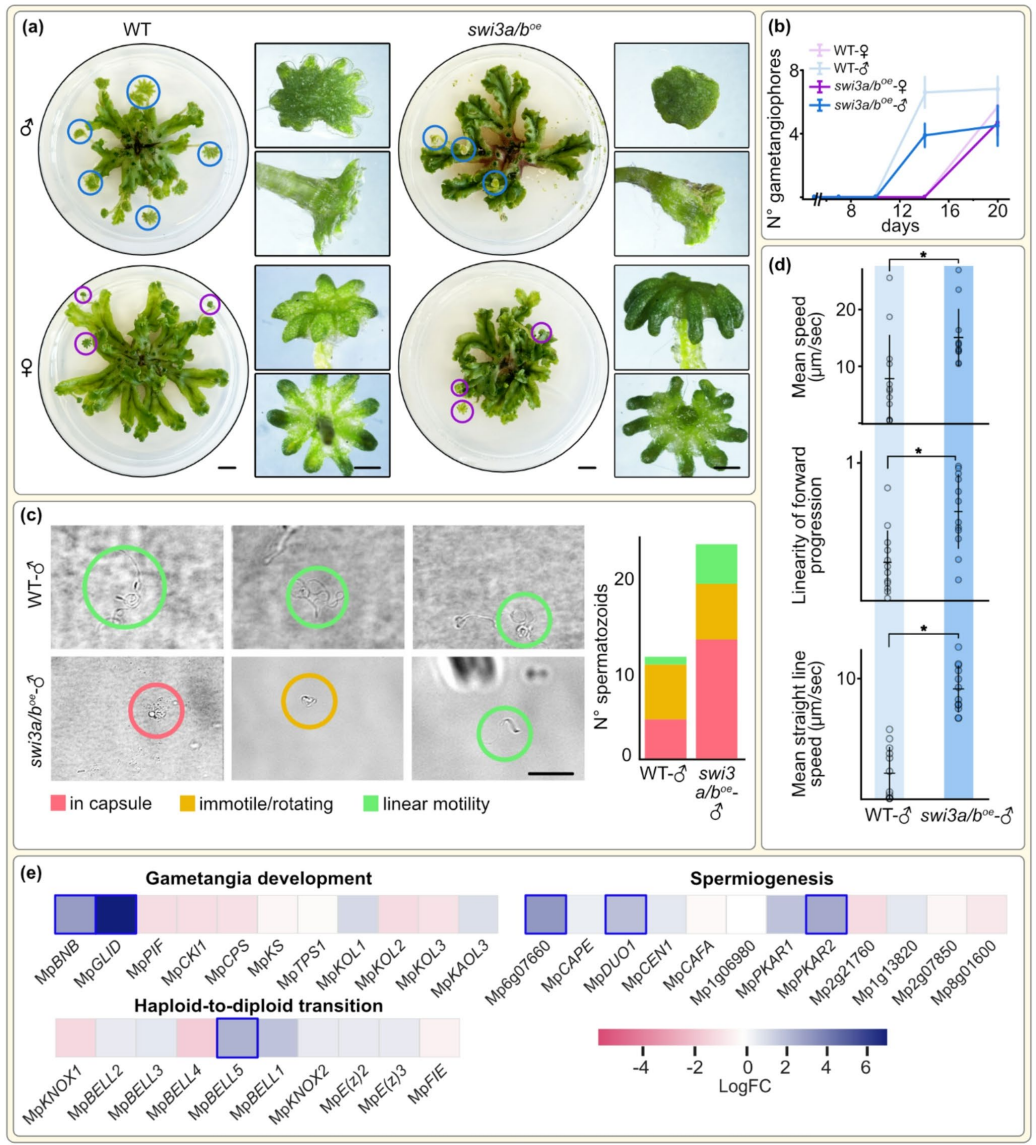
MpSWI3A/B mutants show defective antheridiophore formation and altered spermiogenesis. **(a)** Representative 30 days-old male and female plants from wild-type and *swi3a/b*^oe^ lines. Full view of the thalli with their gametangiophores highlighted by circles, which are shown enlarged in the squares. **(b)** Gametangiophore maturation over time (*n* = 15). **(c)** Representative images of sperms (left) and sperm motility patterns (right) from WT-♂ and *swi3a/b*^oe^*-*♂. Scale bar: 20 μm. **(d)** Mean speed, linearity of forward progression, and mean straight line speed of sperms from WT and *swi3a/b*^oe^-♂ lines (*n* = 10). Statistically significant differences (P value < 0.05) are indicated by asterisks. **(e)** LogFC values for a key subset of genes differentially expressed in *swi3a/b*^*oe*^-♂ as compared to the WT-♂ cultivated under WL supplemented with FR light; genes with significant differences in their expression are marked by a blue outline

To investigate the role of SWI3A/B in the haploid-to-diploid transition and sexual development, we attempted to generate distinct male and female Mp*SWI3A/B* mutants using CRISPR/Cas9(Sugano et al. 2018). 30 candidate lines were analysed per sgRNA utilised. sgRNAs targeting the Mp*SWI3A/B* coding region never yielded transformants with any mutations, but sgRNA targeting the 5’ upstream region provided us with male and female mutants carrying insertions, deletions, or substitutions in their promoter (Fig. S2a). Quantitative real-time PCR on one male and one female mutant showed overexpression of Mp*SWI3A/B* in comparison to the WT (Fig. S2b), which is why we refer to them as *swi3a/b*^oe^-♂ and *swi3a/b*^oe^-**♀** hereafter. Both these lines showed no major defects in vegetative growth (Fig. S3). After two weeks under white light (WL) supplemented with far red light (FR) – which induces the transition to reproductive phase – the WT and *swi3a/b*^*oe*^-♂ both switched to the reproductive stage as evident from antheridiophores formation within twelve days. *Swi3a/b*^*oe*^-♂, however, produced approximately only half as many antheridiophores. In contrast, archegoniophore production in the mutants did not change (Fig. 1a-b). Mutants also showed branching and meristem proliferation different from the WT, which is likely to have contributed to differences in gametangiophore development and number.

Given the role of Mp*SWI3A/B* in antheridiophore development, we examined whether *swi3a/b*^oe^-♂ antheridiophores produced motile sperms. WT spermatozoids matured to stage-5 as expected, whereas those of *swi3a/b*^oe^-♂ frequently appeared to be arrested at earlier stages (Fig. 1c). In WT, 38.5% of sperm remained capsulated, whereas in *swi3a/b*^oe^-♂ this proportion increased to 55.6% (Fig. 1c), underscoring a large (yet at this point statistically insignificant) difference in sperm development. *Swi3a/b3*^oe^-♂ sperms also exhibited a significantly higher straight-line speed, greater linearity of forward progression, and overall increased swimming velocity (Fig. 1d). These results suggested that Mp*SWI3A/B* influences both early spermiogenesis and motility dynamics of mature sperms.

High expression of Mp*SWI3A/B* in antheridia and antheridiophores further corroborated its role in male reproductive development (Fig. S4). We performed comparative RNA-seq analysis of plants grown under WL supplemented with FR light for 14 days. *swi3a/b*^oe^-♂ showed an upregulation of 584 genes and a downregulation of 409 (|log₂FC| > 2) (Fig. S5a, Table S1). Functional annotation of differentially expressed genes revealed enrichment for categories related to plant defence, peroxidase activity, and cell-wall remodelling (Fig. S5b). We next examined genes specifically linked to sexual and asexual reproduction. Among those involved in gametangiophore and gametangia development, Mp*BONOBO* (Mp*BNB*) and Mp*GLID* were strongly upregulated (log₂FC > 3), whereas Mp*PIF*, Mp*CKI1*, Mp*CPS*, and Mp*KOL2/3* were downregulated (Fig. 1e). Genes associated with the haploid-to-diploid transition showed a mixed pattern: Mp*BELL1* and Mp*BELL5* were upregulated, while Mp*KNOX1* and Mp*BELL4* were downregulated. Transcripts linked to sperm differentiation and motility, including Mp*DUO1* (a master regulator of spermiogenesis in land plants), Mp*PKAR* (a regulator of sperm motility) and a flagellar radial spoke protein homolog (Mp6g07660), were upregulated (Fig. 1e, Table S2). These patterns are consistent with the morphology and motility changes observed in *swi3a/b*^oe^-♂ sperms in *Marchantia* (Fig. 1c–d) as well as *Physcomitrium*, where *SWI3A/B* loss of function led to impairment in late maturation (Genau et al. 2021).

### SWI3A/B overexpression reinforces vegetative growth

**Fig. 2:**
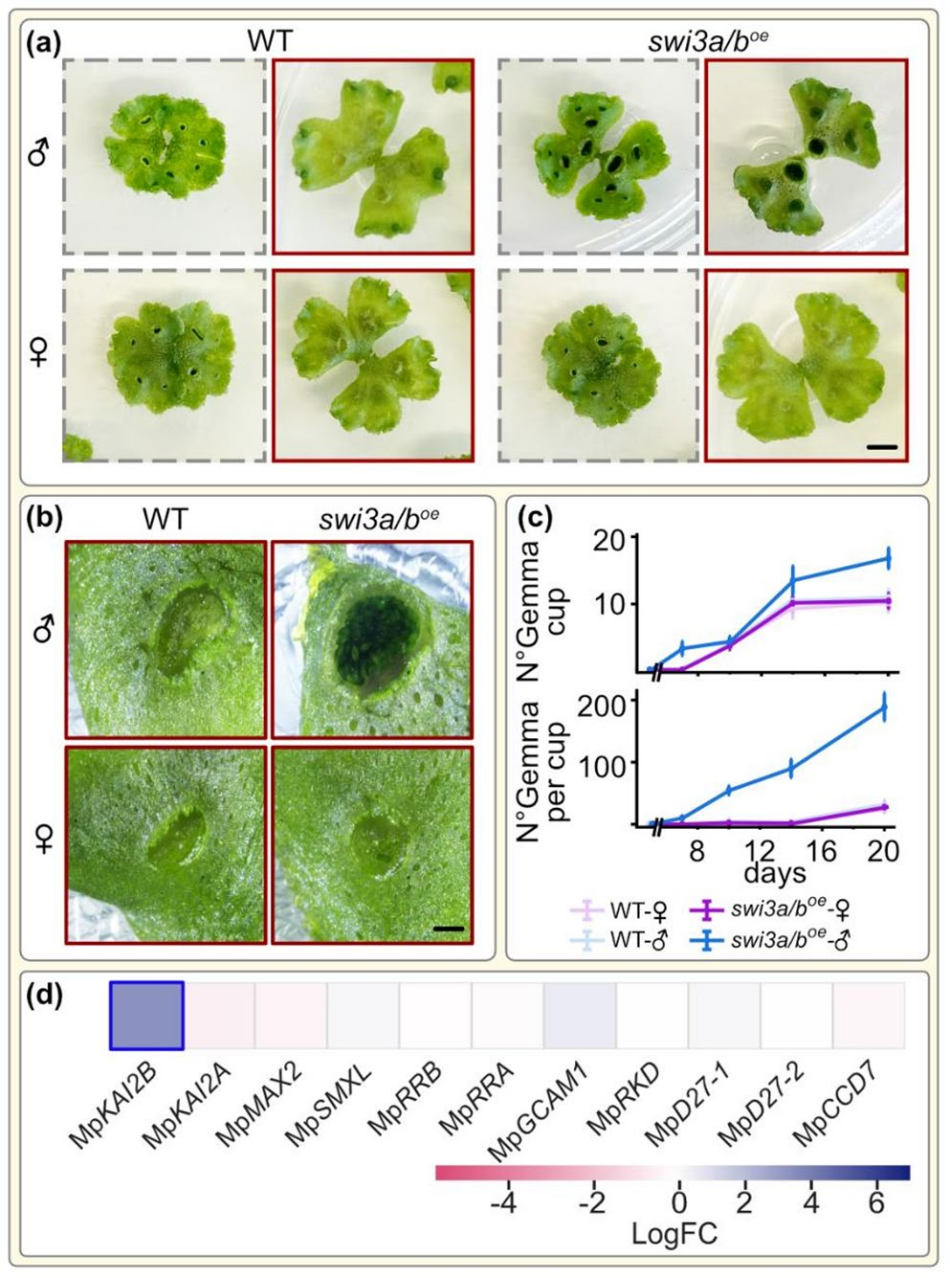
*swi3a/b*^oe^-♂ overproduce gemmae under white light supplemented with far-red light. **(a)** Representative plants of 14d-old wild-type and *swi3a/b*^oe^ lines. Plants outlined with a grey dashed border were grown under continuous white light, whereas those outlined in red were supplemented with far-red light. Scale bar: 0.4 cm. **(b)** Closer view of representative gemmae cups of plants grown with supplemented far-red light. Scale bar: 0.2 cm. **(c)** Gemma cup formation over time and number of gemmae per cup counted; *n* = 15 for thalli and cups analysed. **(d)** LogFC values for key genes involved in gemma formation in *swi3a/b*^oe^-♂ (as compared to the WT-♂) under WL supplemented with FR light. Genes with significant differences in expression are marked by a blue outline

Under FR light, the WT showed a complete reproductive switch, including thallus flattening, gametangiophore formation, and empty gemma cups, whereas *swi3a/b*^oe^-♂ showed a delayed gametangiophore formation. However, *swi3a/b*^oe^-♂ produced significantly more gemma cups (16.2 ± 1,5) than male (10.5 ± 1,2) and female WT (9.7 ± 1.25)(Fig. 2a–b). While WT suppressed gemmae production, *swi3a/b*^oe^-♂ produced 13.5-fold more gemmae at day 10 (Fig. 2). These results suggest that MpSWI3A/B overexpression reinforces asexual reproduction through excessive gemma production under WL supplemented with FR light, while showing a substantial delay in gametangiophore formation and defects in sperm maturation; both trademarks of sexual reproduction. This was associated by expression changes in a number of genes involved in vegetative growth (Fig. 2d). This included the substantial overexpression of MpKAIB, a key gemmae-formation factor contributing to the excessive gemma formation in *swi3a/b*^oe^-♂(Komatsu et al. 2023) (Fig. 2). Moreover, several bHLH transcription factors (MpSETA, MpBHLH29, MpBHLH30, MpBHLH31, MpBHLH21, and MpPIF) were downregulated, whereas late embryogenesis-related genes such as Mp*LEA-like44* and Mp*LEA-like45* were strongly upregulated (Fig. S5c). Together, these transcriptional changes suggest that MpSWI3A/B regulates genes related to sexual differentiation and may also directly or indirectly affect expression of genes related to asexual propagation. We therefore hypothesize that MpSWI3A/B may act as a chromatin-level switch in the liverwort, balancing reproductive and vegetative growth, rather than unidirectional control of reproductive onset.

## Discussion

Sexual reproduction is a defining trait of eukaryotic life, ensuring genetic recombination through the fusion of haploid gametes to form a diploid zygote (Goodenough and Heitman 2014; Speijer et al. 2015; Garg and Martin 2016; Raval et al. 2022; Jeffries et al. 2025). Across eukaryotic lineages, this fundamental process is elaborated by mechanisms that regulate the differentiation, compatibility, and union of gametes – from mating-type systems in unicellular algae and fungi to complex sexual organs in multicellular plants and animals (Heitman 2015; Yadav et al. 2023; Rizos et al. 2024; Becker et al. 2025). The evolution of these mechanisms accompanied the transition from aquatic to terrestrial life, where new developmental structures and regulatory cues ensured gamete encounter and fertilization in the absence of water (Niklas and Kutschera 2010; She and Baroux 2014; Mori et al. 2015; Becker et al. 2025). Among early land plants, bryophytes provide a window into the origin of this reproductive complexity, predating the elaboration of pollen and seeds and offering a distinct perspective on the regulation of the vegetative–reproductive transition (Zhang et al. 2020; Donoghue et al. 2021a, b; Harris et al. 2022; Linde et al. 2023).

Much of our understanding of the regulatory networks governing the transition between vegetative and reproductive phases, however, comes from studies in angiosperms. Here, numerous transcription factors, signalling cascades, and chromatin-remodelling complexes have been characterized (Blazquez & Weigel 2000; Andrés and Coupland 2012; He 2012). A key role for SWI3 is evident in *Arabidopsis*, where it regulates multiple reproduction-related developmental traits, including flowering time and reproductive organ formation (Sarnowski et al. 2005). Loss of AtSWI3A or AtSWI3B results in embryo abortion and male sterility, respectively, while mutations in AtSWI3C or AtSWI3D alter sexual-organ development (Sarnowski et al. 2005). Our findings speak towards a similar role in the bryophyte *Marchantia*, a sister lineage to seed plants.

Sexual reproduction in *Marchantia* is induced through the coordinated action of light-signalling pathways that integrate far-red irradiance and photoperiodic cues (Inoue et al. 2019). Key genes driving the formation of male gamete precursor cells Mp*BNB* and Mp*GLID *(Ren et al. 2024) were significantly upregulated in *swi3a/b*^oe^-♂, yet the plants developed fewer gametangiophores. We speculate that MpSWI3A/B overexpression may cause premature or ectopic activation of these two genes in vegetative tissues, producing – as previously reported (Ren et al. 2024) – a transcriptional signature of reproductive induction without proper organ differentiation. Such an uncoupling between germline specification and organogenesis in *swi3a/b*^oe^*-♂* may reflect impaired FR-phytochrome-PIF signalling and reduced gibberellin (GA) biosynthesis, consistent with the observed downregulation of Mp*PIF*, Mp*CPS*, and Mp*KOL* (Sun et al. 2023). Dedicated research on exactly which cell types show differential expression of these genes and that of meristem morphology will test the uncoupling suggested by our observations and underlying mechanisms. For instance, PIF- and GA-dependent pathways act upstream of the Mp*BNB–*Mp*GLID* module to promote organ initiation and maturation; their downregulation could therefore weaken gametangiophore formation, even when germline markers remain elevated (Sun et al. 2023). Similarly, GA-deficient mutants produce fewer and morphologically abnormal gametangiophores (Sun et al. 2023). Together, these observations let us speculate that *SWI3A/B* overexpression may disrupt the coordination between germline activation and organ differentiation.

Beyond its role in male reproductive development, our results hint at an interesting response to delayed reproductive development caused by excess SWI3A/B, where vegetative propagation through excessive gemmae production is reinforced. A key regulator of gemmae production and vegetative growth is MpKAI2 (KARRIKIN INSENSITIVE2), which drives gemma cup formation and gemma initiation (Komatsu et al. 2023). Strong overexpression of MpKAI2B in *swi3a/b*^oe^-♂ lines underlies reinforcement of vegetative growth. This finding suggests that SWI3 may have a broader role in balancing vegetative and reproductive programs as an epigenetic modulator of phase transitions in the bryophyte. Such a shift toward vegetative propagation, particularly in lines where reproductive growth is compromised, may reflect a bet-hedging strategy in which vegetative reproduction augments survival when sexual reproduction is impaired or delayed (e.g. when conditions are suboptimal for fertilization). Such developmental plasticity might have been crucial for the survival of early land plants adapting to novel terrestrial stresses and continues to contribute to the resilience of extant bryophytes that thrive across diverse habitats.

Altogether, our findings suggest SWI3A/B to be a key chromatin regulator orchestrating the balance between vegetative propagation and sexual reproduction in *Marchantia* males. This is the first report of roles of SWI3A/B during the vegetative stage (before gametaniophore formation), in contrast to other species (Sarnowski et al. 2005; Genau et al. 2021). By modulating the accessibility of key regulatory loci, MpSWI3A/B likely integrates environmental and developmental cues to influence whether cells reinforce vegetative or reproductive fates. On the molecular level, SWI3A/B, via modulation of H3 K27 acetylation, probably acts as a counterpart of PRC2, a writer of H3K27me3 repressive marks (Sarnowski et al. 2005; Zheng and Chen 2011; Wu et al. 2012). Further untangling how SWI3A/B-mediated chromatin remodeling governs these developmental transitions, would reveal the molecular basis of reproductive plasticity in bryophytes and provide evolutionary insight into how chromatin dynamics and diversification of factors such as SWI3A/B facilitated the shift from reversible vegetative–reproductive balance in early land plants to fixed floral commitment in angiosperms. The mechanisms by which chromatin regulation integrates environmental and developmental signals to determine reproductive fate remain poorly understood outside of angiosperms. Bryophytes, particulary dioecious species, provide a powerful model to uncover how these ancestral processes evolved into the complex reproductive strategies of terrestrial plants.

### Limitations of the study

This study investigated the role of SWI3A/B in regulating the transition from vegetative to sexual reproduction in *Marchantia*. Interpretation of the results, however, should take into account some experimental limitations. We generated 30 independent plant lines per sgRNA from three different sgRNAs. Of these 90 independent lines, only four carried mutations and in all cases in the promoter region. We hypothesize that MpSWI3A/B is an essential gene and future studies should hence consider a knock down or conditional knock out and methods such as cre/lox excision. Furthermore, the phenotypes observed in the overexpression lines may be confounded by potential consequences of overabundance (e.g. unspecific interaction with novel proteins) and interfering with the overall function of the SWI complex. As such, it is unclear at this stage, whether the phenotypes are gain- or loss-of-function and how accurately they represent biological roles of MpSWI3. The phenotypes may also be confounded by a non-clonal background of the spores in which mutations were generated, a limitation that remains to be addressed by generating mutations in the thallus stage. RNA-seq could also be conducted from various tissues and developmental stages to obtain a higher resolution. Future studies, including chromatin immunoprecipitation (ChIP-seq), will be essential to identify SWI3A/B target genes and clarify the molecular details of how MpSWI3 acts on sex determination in the bryophyte. Furthermore, genetic crosses evaluating the fertility of swi3a/b^oe^-♂ would help determine, whether SWI3 influences not only reproductive development but also the alternation of generations in *Marchantia*.

## Materials and methods

### Plant growth conditions

*Marchantia polymorpha* (BoGa ecotype; Botanical Garden, Osnabrück University, Germany) was cultivated on half-strength Gamborg´s B5 medium containing 1% glucose and 1% agar under continuous full light spectrum (450–700 nm) 70 µmol m^− 2^ s^− 1^ at 22 °C. To induce the reproductive stage, the light conditions were supplemented with far-red light (700–750 nm) 40 µmol m^− 2^ s^− 1^ at 22 °C in ECO^2^ boxes (oval, 80 mm) with a green filter #40 (Duchefa Biochemie).

### Generation of mutant plants

CRISPR/Cas9-based genome editing of MpSW3A/B (Mp8g15610) was performed as previously described (Sugano et al. 2018). Three sgRNAs (sgRNA1,2,3) were designed, sgRNA1 targeted the promoter region, sgRNA2 and sgRNA3 targeted the coding sequence of Mp*SWI3A/B* (Fig. S2a). Cloning of sgRNA was performed using pMpGE_En03 as the entry- and pMpGE010 as a destination vector using LR Clonase II enzyme mix (Thermo Fisher Scientific). Destination vectors were introduced into electrocompetent *Agrobacterium tumefaciens* (GV301 without pSOUP) by electroporation (Bio-Rad GenePulser Xcell, 1.44 kV). *Agrobacterium*-mediated sporeling transformation was performed as described previously (Ishizaki et al. 2008). After three days of spore co-cultivation with *Agrobacterium* transfectants, sporelings were washed three times with liquid ½ Gamborg´s B5 medium to remove Agrobacteria and plated on agar plates with half-strength Gamborg´s B5 medium with 1% Glucose supplemented with 10 µg/mL Hygromycin and 100 µg/mL Cefotaxime for selection.

### Macroscopic phenotyping

To assay gametangiophore numbers, 15 plants per line were grown in ECO^2^ boxes that allow the upwards growth of gametangiophores. Each gametangiophore and gemma cup was counted as soon as it could be seen by sight from the top of the thallus. To quantify gemmae per cup, all gemmae from gemmae cups closer to the thallus base were harvested by a toothpick, gemmae were spread on a plastid sheet, imaged and counted using ImageJ(1.54f).

### Sperm phenotyping

Sperms were harvested by applying water on top of the antheridia from 30 days old plants cultivated under WL supplemented with FR light. After 5 min of incubation, the water was collected and sperm morphology monitored with a Nikon eclipse Ti imaging platform. For quantitative motility analysis, sperms were video-recorded at the resolution of 1280 × 1024 pixels and at the rate of 33 frames per second (fps) by a Nikon Digital Sight DS-U3. Movies were converted into a sequence of individual frames and sperm movement tracked by the ImageJ plugin TrackMate v7.11.1. Two movies were analyzed to obtain sperm swimming motility parameters for each line.

### RNA-seq

Total RNA was extracted from WT and *swi3a/b*^oe^-♂ plants grown from gemmae under WL supplemented with FR light for 14 days, using the RNeasy Plant Mini Kit for RNA Extraction (Qiagen). Whole thalli without any visible gametangiophores were harvested. Three biological replicates were prepared for each genotype and mRNA isolated via their poly(A) tail. RNA integrity and concentration were assessed using a NanoDrop spectrophotometer. Libraries were prepared by Eurofins Genomics (Ebersberg, Germany). Sequencing was performed on an Illumina NovaSeq 6000 platform, generating paired-end reads (2 × 150 bp), with an average of ~ 30 million read pairs per sample. Sequencing data was quality checked and analyzed as described previously (Frangedakis et al. 2024). Briefly, high quality reads were retained using FASTQC and TrimGalore, aligned to *Marchantia polymorpha* genome (v.7.1) using Kallisto (Bray et al. 2016; Bowman et al. 2017) and DEG were obtained through DESeq2 (Love et al. 2014).

## Supplementary Information

Below is the link to the electronic supplementary material.

Supplementary Material 1

Supplementary Material 2

Supplementary Material 3

Supplementary Material 4

Supplementary Material 5

Supplementary Material 6

Supplementary Material 7

Supplementary Material 8

Supplementary Material 9

## Data Availability

Supplementary figures and tables are available with this submission. Transcriptome data are available in the NCBI Sequence Read Archive: PRJNA1353859.
